# Interleukin-6 c.-174G>C Polymorphism and Periodontitis in a Brazilian Population

**DOI:** 10.1155/2014/490308

**Published:** 2014-12-04

**Authors:** Fernanda Gabriela Teixeira, Samir Andrade Mendonça, Kamilla Menezes Oliveira, Djanilson Barbosa dos Santos, Lucas Miranda Marques, Maise Mendonça Amorim, Raquel de Souza Gestinari

**Affiliations:** ^1^Laboratory of Cellular and Molecular Biology, Multidisciplinary Health Institute, Federal University of Bahia, 45029-094 Vitória da Conquista, BA, Brazil; ^2^Health Sciences Center, Federal University of Bahia Recôncavo, 44380-000 Santo Antonio de Jesus, BA, Brazil; ^3^Laboratory of Microbiology and Immunology, Multidisciplinary Health Institute, Federal University of Bahia, 45029-094 Vitória da Conquista, BA, Brazil; ^4^NUPEM, Federal University of Rio de Janeiro, Avenida São José do Barreto, 764 São José do Barreto, 27965-045 Macaé, RJ, Brazil

## Abstract

*Aim*. Periodontitis is an inflammatory disease that affects the teeth supporting structures, triggered by periodontal pathogens, and is influenced by environmental and genetic factors. Genes encoding molecules related to the immune response, such as cytokine, are the main candidates for polymorphisms analysis and may be possibly associated with this pathology. A G/C promoter polymorphism on the *IL6* gene has been shown to affect basal IL-6 levels. The aim of this study was to investigate the association between the *IL6* c.-174G>C polymorphism and periodontitis in individuals from Vitória da Conquista, Bahia, Brazil. *Material and Methods*. Three hundred and thirty individuals (134 cases, 196 controls) were genotyped for the *IL6* c.-174G>C by MS-PCR technique. Concentrations of salivary IL-6 were determined by ELISA method. *Results*. The *IL6* c.-174G>C polymorphism was associated with periodontitis when comparing the distribution of genotypes between patients with periodontitis and control subjects. The GC genotype appeared as a protective factor for periodontitis. Results showed increased levels of salivary IL-6 in periodontitis patients. Nevertheless, there was no relationship between the concentrations of IL-6 and genotypes when comparing the case and control groups. *Conclusions*. Our data indicate an association between *IL6* c.-174G>C polymorphism and periodontitis and showed that IL-6 may be considered an important marker for periodontitis.

## 1. Introduction

Periodontal disease is an inflammatory condition brought about by the interaction between microorganisms that compose supragingival and subgingival biofilms and the host inflammatory response [1]. Some of these microorganisms have been shown to be responsible for the periodontal disease initiation and progression, and the red complex, which includes* Porphyromonas gingivalis*,* Treponema denticola,* and* Tannerella forsythia, *is considered the most important pathogens in periodontal disease [2, 3].

The inflammation progression induces the production of persistent inflammatory signals by the cells of periodontal tissues. Such persistent signals, represented by the proinflammatory cytokines, for example, interleukin-1 (IL-1), interleukin-2 (IL-2), and tumor necrosis factor-alpha (TNF-*α*), stimulate the production of secondary mediators which in turn amplify the inflammatory cascade. This continuing amplification process results in the increased production of proteases and osteoclastic signals that lead to connective tissue and bone destruction [4, 5].

Although the host immune response is caused by bacterial infection, the inflammatory response of periodontal tissues is influenced by environmental and genetic factors [6]. Smoking, hygiene habits, age, educational level, and systemic diseases (e.g., diabetes and osteoporosis) are considered risk factors for the prevalence, extent, and severity of periodontal disease [7–9]. Regarding the genetic factors, genes that play an important role in the immune pathology of periodontal disease such as cytokine genes are the main candidates for the analysis of polymorphisms and may be possibly associated with periodontal disease [10]. These polymorphisms may cause a change in the encoded protein, or its expression, possibly resulting in alterations in innate and adaptive immunity, and may thus be deterministic in disease outcome [11, 12].

One of the well-studied cytokines genes whose variants are associated with periodontal disease occurrence is interleukin-6. IL-6 has diverse functions, including differentiation and activation of macrophages and T cells and growth and terminal differentiation of B cells. The control of IL-6 levels plays a crucial anti-inflammatory role in both systemic and local inflammatory responses [13]. Individual variations in the blood levels of IL-6 may modulate the predisposition to a number of inflammatory diseases, such as periodontitis, because it is a proinflammatory mediator that activates host cells and may in turn lead to extracellular matrix destruction. Such individual variations seem to be regulated by common nucleotide variations in the IL6 encoding gene, located on chromosome 7p21 [14].

Association between* IL6* gene polymorphisms and periodontitis in different ethnicities has been widely investigated and the results suggest that* IL6* c.-174G>C polymorphism is associated with periodontopathogens detection, variations in severity of inflammation, tissue destruction, and attachment loss in periodontal disease [15, 16]. Therefore, the aim of the present study was to investigate the association between* IL6* c.-174G>C gene polymorphism and periodontitis in a sample from Bahia, Brazil.

## 2. Material and Methods

### 2.1. Ethical Considerations

The study protocols were reviewed and approved by the Research Ethics Committee of the State University of Southwest of Bahia (Protocol number 071/2009). Written informed consent was obtained from all participants before starting the study.

### 2.2. Study Population and Clinical Examination

From March 2010 to August 2012, a total of 330 residents of Vitória da Conquista, Bahia, Northeast Brazil, aged between 14 and 69 years of both genders, were recruited to this study. The number of the sample was representative according to the sample size calculation. The subjects were submitted to anamnesis and received a complete periodontal examination including supragingival/subgingival calculus, bleeding on probing (BOP), probing depth (PD), and clinical attachment loss (CAL). The diagnostic criteria for periodontitis were referred to the 1999 International Classification of the Periodontal Disease and Conditions [17]. Individuals were excluded from the study if they (i) were pregnant or lactating women, edentulous, or children; (ii) had received antibiotic treatment in the previous 3 months; and/or (iii) were taking long-term anti-inflammatory or immunosuppressive drugs. After diagnostic phase, our sample was composed of 134 patients with chronic periodontitis ((28 males and 106 females) − (47.5 ± 27.58, mean ± SD)) and 196 periodontally healthy subjects (control group) ((42 males and 154 females) − (33.5 ± 19.09)).

### 2.3. Sample Collection

Epithelial cells were obtained from all subjects for genomic DNA extraction. The sampling was made by scraping the oral mucosa using a custom-made sterile wooden spatula, which was then immediately immersed in a 2 mL sterile microtube. For IL-6 levels determination, individual saliva samples (5 mL) were collected by expectorating into disposable tubes. The samples were stored at −20°C until assayed. Saliva was not stored longer than 2 months.

### 2.4. Isolation of Genomic DNA and IL6 c.-174G>C Genotyping

Genomic DNA was extracted with an alkaline solution as previously described [18]. Extracted DNA was labeled and stored at −20°C until further genotyping. Genotyping of the c.-174G>C* IL6* promoter polymorphism was performed by the mutagenically separated polymerase chain reaction (MS-PCR) [19] using specific primers described by Schotte et al. [20]. Amplification was performed on a Longene thermocycler in a 25 *μ*L reaction mixture which included 100 *η*g of genomic DNA solution, 1 U of* Taq* DNA polymerase (Invitrogen Life Technologies, São Paulo, Brazil), and primers in the following concentrations: 5 *ρ*mol IL-6G; 15 *ρ*mol IL-6C; 3 *ρ*mol IL-6 complementary strand primer. Thermal cycling conditions were initial denaturation at 94°C for 10 min, followed by 45 cycles of amplification at 94°C for 20 s and annealing at 57°C for 1 min 20 s, with extension for 1 min 20 s at 72°C, with a final extension for 5 min at 72°C. The specificity of MS-PCR for the different genotypes was confirmed by direct sequencing of genomic DNA (data not shown).

### 2.5. Electrophoresis and Visualization of MS-PCR Products

Aliquots (12 *μ*L) of the MS-PCR products were separated by size on 4% agarose gels stained with ethidium bromide (0.5 *μ*g/mL), visualized under ultraviolet (UV) light (Transilluminator) and photographed at appropriate photodocumentation system (LABImage L-PIX (H.E.), Loccus Biotecnologia, Brazil). PCR products that were 121 base pairs (bp) (*G* allele) and 136 bp (*C* allele) in length could be distinguished readily.

### 2.6. Determination of IL-6 Levels

The IL-6 levels were measured using the whole saliva samples by the enzyme-linked immunosorbent assay (ELISA) according to the manufacturer's instructions (eBioscience, San Diego, CA, USA). The 96-well microplates were read at 450 *η*m wavelength using Vivid Vision Microplate Reader (ALKA Tecnologia, São Paulo, Brazil). The levels of IL-6 in the samples were obtained by comparison with the standard curve generated from the standards supplied by the manufacturer.

### 2.7. Statistical Analysis

The magnitude of the associations between risk factors and periodontitis was estimated by the odds ratio (OR) with a 95% confidence interval (95% CI). The Chi-square test (*χ* ^2^) measured the association between the independent (age, gender, scholarship, smoking, diabetes, cardiorespiratory diseases, tooth mobility, gingival bleeding, gingival sensitivity, and discomfort when chewing) and dependent (periodontitis) variables. All variables with *P* < 0.20 in the *χ* ^2^ were included in multivariate analysis using logistic regression. A backward deletion strategy was applied, and those variables with *P* values equal to or less than 0.05 remained in each of the final models. Goodness of fit was evaluated using the Hosmer-Lemeshow test, while the Stata software version 10.0 was used for data analysis. (Stata Corp., College Station, USA).

Allelic and genotypic frequencies were obtained by direct counting. Contingency tables were used with Chi-square tests to compare observed genotype frequencies with those expected under Hardy-Weinberg equilibrium. Comparison between groups was made using Chi-square and Fisher's exact tests for other categorical variables. The Kruskal-Wallis test for independent samples was used to compare nonparametric data. Associations with *P* value <0.05 were considered significant.

## 3. Results

In this study, 330 individuals were analyzed regarding the periodontal health, possible risk factors, and more frequent symptoms common to periodontitis.  Table 1 summarizes the basic sample characteristics and the overall results of the periodontal examination and anamnesis. A positive association between the 30-year or older patients (OR = 2.54; 95% CI: 1.60–4.04), lower educational level (OR = 1.67; 95% CI: 1.03–2.72), and smoking (OR = 9 2.20; 95% CI: 1.19–4.07) was found. The other factors such as gender, diabetes, and cardiovascular disease showed no significant association with the periodontitis occurrence in the population. Regarding the symptoms involved in the absence of periodontal health, a positive association between periodontitis and tooth mobility (OR = 3.05; 95% CI: 1.81 to 5.13) and discomfort when chewing (OR = 1.83; 95% CI: 1.17 to 2.87) was found.

In Table 2, the odds ratio (OR) for periodontitis with their respective confidence intervals (CI 95%) estimated by multivariate logistic regression model is presented. In multiple logistic regression, age equal or superior to 30 years, smoking, and tooth mobility were associated with a higher risk of having periodontitis.

The selected sample was evaluated for the periodontal disease association with* IL6 *c.-174G>C gene polymorphism. The genotype distribution in the control group fulfilled Hardy-Weinberg criteria (*χ* ^2^ = 0.05; *P* = 0.82). There was a significant difference in the genotypic frequency distribution when comparing the control subjects with periodontitis patients (*P* = 0.038), showing an association between this polymorphism and periodontitis (Table 3).

The genotypes distribution in the total population sample was 75% for the GG genotype, 21% for GC genotype, and 4% for the CC genotype. The* G *allele frequency was 86%, while the* C* allele was 14%. In order to verify the association with periodontitis, the frequencies of each genotype for the* IL6* c.-174G>C polymorphism were compared in case and control groups. The GC genotype appeared as a protective factor for periodontitis (Table 4).

**Figure 1:**
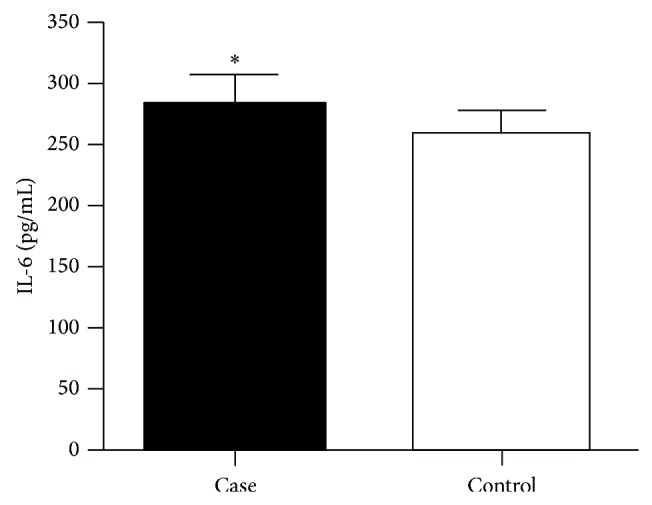
IL-6 salivary dosage in saliva of individuals in the case and control groups using ELISA. Analysis performed by Mann-Whitney (*P* < 0.05). ^*^ *P* < 0.05.

The local expression of IL-6 and its relation with the* IL6* c.-174G>C polymorphism and periodontitis was also analyzed. Significant differences in the concentration of salivary IL-6 between case and control groups were observed (*P* = 0.038) (Figure 1). Our findings showed that the IL-6 increased levels in the case group indicate that the higher production of this cytokine is associated with periodontitis and its inflammation response.

**Figure 2:**
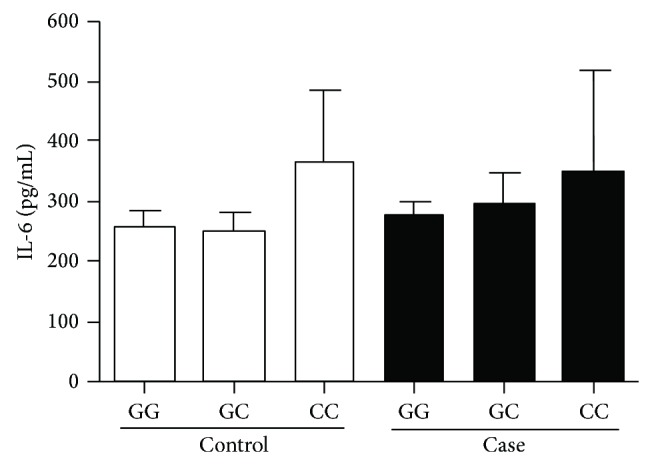
Quantification of IL-6 cytokine in the saliva of individuals of the case and control groups separated by c.-174G>C polymorphism genotype, using ELISA. Analysis performed by Kruskal-Wallis.

IL-6 expression was compared with different genotypes for the* IL6* c.-174G>C polymorphism, separating them into case and control groups (Figure 2). A significant difference was observed concerning IL-6 levels in the studied population (*P* = 0.0099). Observing the protein expression for each genotype, it was found that the CC genotype was associated with increased production of IL-6, but there was no significant difference when comparing the IL-6 quantification among the different genotypes and the case and control groups. Thus, higher salivary concentrations of IL-6 observed in patients with periodontitis may be related to other factors than the genetic influence, especially environmental risk factors, such as bacterial load or poor oral hygiene habits.

## 4. Discussion

In the studied population, the periodontitis was more prevalent in individuals older than 30 years, in subjects from the lower educational strata, and in those who were using tobacco regularly. The identification of risk factors associated with periodontal disease in different populations enables the implementation of effective interventions for health promotion. Public policies that act for the prevention of periodontitis, with the realization of preventive antismoking campaigns and intensified action in individuals older than 30 years, can minimize the influence of these risk factors and hence reduce the prevalence of the disease in the studied population.

Age has been identified in several studies as an important risk factor for periodontitis. Haas et al. [21] showed that, in a population of southern Brazil, the loss insertion due to the progression of periodontitis was more prevalent in individuals older than 30 years. The results of this study suggest that the consequences of the development and progression of periodontitis are more prevalent in older individuals and corroborate the results obtained by other authors [22, 23], justifying, at least in part, the increase of tooth loss in the older age groups [24–26]. Nevertheless, other authors state that periodontal disease should not be considered a natural event of aging and that this association is a result of the cumulative nature of the disease [27, 28].

Smoking is considered, by several authors, the main risk factor for prevalence, extent, and severity of periodontal disease [8, 29]. Thomson et al. [30] assessed the prevalence of periodontitis in three different age groups and showed that the disease begins in adulthood and its progression is accelerated with age, but in smokers the process evolves quickly with advanced dental insertion loss, independent of range age. However, smoking was not associated with tooth loss in the Sri Lankan population [31].

The educational level has also been evaluated as a risk factor for periodontitis, and low education has often been associated with it [30, 32, 33]. In the subjects recruited for this study, the disease was more prevalent in individuals who had not completed high school, with a maximum of nine years of education. In another Brazilian population sample, the risk of dental insertion loss due to disease progression increased by 53% in individuals who studied four years or less, compared to those with higher education [21]. Buchwald et al. [34] showed that the prevalence of periodontitis in a sample from Germany is related to socioeconomic status and educational level being the determinant factor for the disease.

All risk factors can still be added to local infection and result in inflammation of the tissues supporting the teeth in periodontitis, culminating in symptoms such as bleeding gums, formation of periodontal pockets, and alveolar bone resorption, with varying degrees of dental insertion loss [35, 36]. The tooth mobility due to the insertion loss and the discomfort in chewing were considered clinical factors related to periodontitis in the studied population. The gingival sensitivity and bleeding can demonstrate clinical relationship with periodontitis, but it was not statistically significant. Gingival bleeding may be associated with other diseases of the oral cavity beyond periodontitis, as gingivitis, which also presents an inflammatory process, however mild and reversible [37, 38]. Our findings can be explained by the high number of patients with gingivitis in the control population in this study, so that the bleeding and gingival sensitivity variables have not shown statistically significant difference among the groups, since these clinical factors are common to both diseases.

The data relating the polymorphism and cytokine expression may contribute to the identification of genetic factors that play a significant role in the etiology of this disease and confirm the diagnosis and determine the susceptibility of individuals regarding periodontal disease in this population. In the present study, a positive association was found between* IL6 *c.-174G>C gene polymorphism and periodontitis, considering the frequency distribution of GG, GC, and CC genotypes among the case and control groups (*P* = 0.038). The GC genotype showed statistically significant relationship with periodontal disease when compared with the other genotypes. Our results are consistent with findings in many different populations [39, 40].

In agreement with our findings, Kalburgi et al. [13] found a significant difference in genotype distribution between cases and controls, showing an association between the polymorphism and periodontitis in Indian population sample. Similarly, Scapoli et al. [40] reported an association of the* IL6* c.-174G>C polymorphism and periodontal disease in a study of Italian population. However, this study determined that the* G* allele is one that has a statistically significant association with periodontal disease.

Brett et al. [39], in an analysis of a population-based sample from London, showed that the GC genotype had greater representation in subjects with chronic periodontitis (42%) when compared with subjects with aggressive periodontitis (26.5%) and control subjects (19.2%) and further suggested that this genotype was associated with an increased risk for periodontal disease. Nevertheless, in this study, the GC genotype was revealed as a possible protective factor for periodontitis, since it was observed significantly more in individuals from the control group. Thus, the results of the studies indicate that the absence of a single genotype for the polymorphism* IL6* c.-174G>C, which acts as a preponderant factor in comparison to others regarding the disease, may suggest that other factors, especially ethnic and/or demographic, may be influencing the relationship between genotype and periodontitis.

In contrast to our data, whose GC genotype was associated with periodontitis, studies have found different results concerning genotypes associated with the disease. The GG genotype was associated with chronic periodontitis in the study conducted in a sample of Brazilian elderly women and in a study with Brazilian Caucasian population [4, 41, 42]. Franch-Chillida et al. [42] described an association of the GG genotype and periodontitis analyzing samples from nonsmokers and those older than 45 years from India. Babel et al. [43], in a study carried out in England, observed that the number of patients with the CC genotype was significantly higher among patients in the case group (41.9%) when compared to controls (27.6%).

There are also studies indicating no association between the polymorphism* IL6* c.-174G>C and periodontal disease. Fan et al. [44] observed a very low prevalence of the* C* allele in a study in a Chinese population and found no significant difference in the genotypes distribution for the c.-174G>C polymorphism in the analysis of groups with periodontal disease and coronary artery disease. Similarly, Holla et al. [45] found no significant differences between patients with periodontitis and healthy controls when they evaluated a Caucasian population sample from Czech Republic regarding the same polymorphism. The data of Loo et al. [46] showed no association between genetic polymorphisms in* IL6*,* IL1*β*,* and* IFNγ* genes and periodontal disease in a study carried out in China. However, in the same study, associations with polymorphisms in* IL1* *α*,* TNF* *α*,* IL4,* and* IL10* genes were described. A study conducted by Wohlfahrt et al. [47] also revealed no association between* IL6 *c.-174G>C polymorphism and other polymorphisms in* CTLA-4 DEFB1, ICAM-1, FasL, ICOS, CCR5, OPG,* and* OPN* genes in subjects with periodontal disease from USA. These data are in disagreement with those obtained in our study.

Although controversial results have been observed in the analysis of the association between* IL6* c.-174G>C gene polymorphism and periodontitis in different population samples, studies indicate that genetic variation may also result in differences in the expression of the cytokine IL-6 according to the genotype which could culminate in changes in the individual inflammatory response [48, 49]. Measurement of molecular markers in salivary fluid has been suggested as a practical screening tool for diseases of the oral cavity, indicating the importance of quantification of proteins related to the immune response [50]. The dosage of the cytokine IL-6 in saliva held in the sample revealed a statistically significant difference in expression between patients with periodontitis compared to those with evidence of periodontal health. However, there was no significant difference in the local expression of IL-6 between case and control groups when we compared the different genotypes for* IL6* c.-174G>C, even if a higher expression of the protein has been observed among patients with disease.

The data in the literature regarding the quantification of IL-6 in salivary fluid, the polymorphism c.-174G>C, and periodontitis are scarce. These findings are in agreement with another report by Loo et al. [46] who demonstrated a significant difference in the expression of IL-6 among Chinese subjects with periodontitis compared to controls. Similarly, Sharma et al. [50], in a study conducted in India, showed an elevation of IL-6 salivary concentrations in patients with leukoplakia with coexisting periodontitis and subjects with periodontitis, compared to the healthy control group.

Concerning the association of the protein dosage and the different genotypes, Smith et al. [51] showed that serum concentrations of IL-6 in patients after intensive periodontal therapy were not associated with genotypes when examining* IL6*-174/-572 polymorphisms, similar to the findings of the present study. Therefore, we believe that the observed higher levels of IL-6 in saliva of patients with periodontitis may be related to other factors prevalent to the analyzed polymorphism. Among these factors, an exacerbated inflammatory response especially stands out due to both environmental risk factors, such as bacterial load, poor oral hygiene habits, and smoking and other genetic factors that are not assessed in our work. The mechanism of multifactorial inheritance described for periodontitis justifies such results, which by definition is a consequence of variations in multiple genes added to the effects of the environment, collaborating together to the risk of developing the disease. The small number of subjects with the CC genotype in the population studied may also be a factor which can explain this result. Nevertheless, Badiu et al. [52] determined that the quantification of IL-6 is important to support the diagnosis and the severity of periodontitis.

This paper provides information for a better understanding of the complex interactions between the host immune response, microorganisms, and genetic factors in individuals with periodontitis. However, we believe that the results obtained regarding the* IL6 *c.-174G>C gene polymorphism can still be added to the analysis of other factors related to the etiology of periodontitis not evaluated here, including other genetic markers, which could provide us a better result on these interactions, and this is a possible limitation of our study.

## 5. Conclusions

Our results indicate an association between* IL6 *c.-174G>C polymorphism and periodontitis and showed that IL-6 may be considered as an important marker for periodontitis. Further studies in other populations are necessary to confirm these findings and determine the role of this polymorphism in the pathogenesis of periodontitis, as well as the association of the disease with the presence of periodontal pathogens and other genetic variations which could be related to the disease.

## Figures and Tables

**Table 1:** Clinical and demographic profile of the group case and control subjects regarding the periodontitis in the analyzed population sample from Vitória da Conquista, Brazil.

Variables	Periodontitis case	Periodontitis control	Odds ratio (raw)	CI 95%	*P* value^*^
	*N* = 134	*N* = 196			
	*n* (%)	*n* (%)			
*Demographic data *					
Age					
≥30 years	95 (70.9%)	100 (51.0%)	2.54	[1.60; 4.04]	<0.001
<30 years	39 (29.1%)	96 (49.0%)	1.00		
Gender					
Female	106 (79.1%)	154 (78.6%)	1.03	[0.60; 1.77]	0.907
Male	28 (20.9%)	42 (21.4%)	1.00		
Scholarship^1^					
<high school	95 (73.6%)	122 (62.6%)	1.67	[1.03; 2.72]	0.038
≥high school	34 (26.4%)	73 (37.4%)	1.00		
Smoking					
Yes	28 (20.9%)	21 (10.7%)	2.20	[1.19; 4.07]	0.011
No	106 (79.1%)	175 (89.3%)	1.00		
Diabetes^2^					
Yes	5 (3.8%)	3 (1.5%)	2.49	[0.58; 10.59]	0.203
No	128 (96.2%)	191 (98.5%)	1.00		
Cardiorespiratory diseases^3^					
Yes	29 (21.6%)	40 (20.6%)	1.06	[0.62; 1.82]	0.823
No	105 (78.4%)	154 (79.4%)	1.00		
*Signals and symptoms *					
Tooth mobility^4^					
Yes	49 (36.6%)	31 (15.9%)	3.05	[1.81; 5.13]	<0.001
No	85 (63.4%)	164 (84.1%)	1.00		
Gingival bleeding					
Yes	90 (67.2%)	120 (61.2%)	1.29	[0.82; 2.05]	0.271
No	44 (32.8%)	76 (38.8%)	1.00		
Gingival sensitivity					
Yes	110 (82.1%)	151 (77.0%)	1.37	[0.79; 2.37]	0.268
No	24 (17.9%)	45 (23.0%)	1.00		
Discomfort when chewing^5^					
Yes	77 (58.8%)	84 (43.8%)	1.83	[1.17; 2.87]	0.008
No	54 (41.2%)	108 (56.2%)	1.00		

Observation loss: ^1^scholarship: 6 losses (*n* = 324); ^2^diabetes: 3 losses (*n* = 327); ^3^cardiorespiratory diseases: 2 losses (*n* = 328); ^4^tooth mobility: 1 loss (*n* = 329); ^5^discomfort when chewing: 7 losses (*n* = 323).

^*^Chi-square (*χ* ^2^) and Fisher's exact tests.

**Table 2:** Factors associated with periodontitis in the analyzed population sample from Vitória da Conquista, Brazil.

Variables	Adjusted odds ratio (CI 95%)			*P* value	
Age					
≥30 years	2.63 (1.59; 4.35)			<0.001	
Smoking					
Yes	2.35 (1.18; 4.67)			0.015	
Tooth mobility					
Yes	2.42 (1.37; 4.28)			0.002	

*Note*. ^*^The adjusted analysis contains the variables with *P* values <0.20 of Table 1: age, scholarship, smoking, tooth mobility, and discomfort when chewing.

**Table 3:** Allelic and genotypic frequencies observed in case and control subjects regarding the *IL6 *c.-174 G>C gene polymorphism.

Genotypes	Periodontitis case		Periodontitis control		*P* value^*^
	*N* = 134		*N* = 196		
	*n* (%)		*n* (%)		
GG	102 (76.1%)		136 (69.4%)		0.038
GC	23 (17.2%)		54 (27.6%)		
CC	9 (6.7%)		6 (3.1%)		

	G	C	G	C	
Allele	0.85	0.15	0.83	0.17	0.598

^*^Chi-square (*χ* ^2^) and Fisher's exact tests.

**Table 4:** Genotypic profile in the group case and control subjects regarding the periodontitis in the population sample.

Variables	Periodontitis case	Periodontitis control	Odds ratio	CI 95%	*P* value^*^
	*N* = 134	*N* = 196			
	*n* (%)	*n* (%)			
*IL6*-dominant model					
GG	102 (76.1%)	136 (69.4%)	1.00		
GC+CC	32 (23.9%)	60 (30.6%)	1.41	[0.85; 2.32]	0.180
*IL6*-codominant model					
GG	102 (76.1%)	136 (69.4%)	1.00		
GC	23 (17.2%)	54 (27.6%)	0.57	[0.33; 0.99]	0.043
CC	9 (6.7%)	6 (3.1%)	2.00	[0.69; 5.80]	0.194
*IL6*-recessive model					
GG+GC	125 (93.3%)	190 (96.9%)	1.00		
CC	9 (6.7%)	6 (3.1%)	2.28	[0.79; 6.56]	0.117

^*^Chi-square (*χ* ^2^) and Fisher's exact tests.
